# 

**Published:** 1988-11

**Authors:** 


Established 1883

### BRISTOL MEDICO-CHIRURGICAL JOURNAL

**NOVEMBER 1988**
**Volume 103 (iv)**

Copyright © Clinical Press Ltd 1988.

**Editor**
Mr M. G. Wilson
*40 Coombe Lane, Bristol BS9 2BJ*
*Tel: 0272-682320*

**Editorial Committee**
Dr Paul Goddard (Assistant Editor)
Mr B. P. Jones (Secretary)
Dr J. L. Burton

**Correspondents**
Dr A. E. Adam (Taunton)
Mr D. C. C. Bartolo
Mr M. L. Crosfil (Penzance)
Dr J. D. Davies
Dr J. Jancar
Professor D. J. Pereira Gray (Exeter)
Dr H. G. Mather
Professor G. M. Stirratt
Mr C. Teasdale (Plymouth)
Dr J. L. G. Thomson
Dr M. J. Whitfield
Professor G. K. Wilcock

BRISTOL MEDICO-CHIRURGICAL SOCIETY

Mr A. John Webb (President)

Mr R. N. Baird (Secretary)
*23 Old Sneed Park, Bristol BS9 1RG*

Dr N. C. Tricks (Treasurer)
*The Mill, Wrington, Bristol*

**Published by**
**Clinical Press,**
*15 Bucklands Drive*
*Nailsea*
*Bristol*

**Typesetting by**
*Avon Communications Ltd.,*
*Avon House, Nailsea, Bristol BS19 2DJ*

**Printed by**
*Suttons Print, Paignton*

**Contents**

*Scire est nescire, nisi id me alius sciret.* **Lucilius (180–102 BC)**

### THE MEDICAL JOURNAL OF THE SOUTH WEST

**NOTICE TO CONTRIBUTORS**
Contributions are invited especially from West of England authors on a variety of subjects, e.g. Original articles relating to the practice of medicine, reports on the contemporary medical scene, obituaries, historical accounts, recollections of colleagues and events worthy to be remembered and liable to be forgotten. An article of 2,000 words with 2 illustrations will occupy 2 pages and this is a suggested, though by no means an obligatory length. Articles are preferred in the form proposed by the International Committee of Medical Editors (Vancouver System)—pamphlet obtainable from Editor of BMJ., BMA. House, Tavistock Square, London, WC1H 9JR.

### INDEX to Volume 103, nos i, iii and iv 1988.

Volume 103 ii, the MRI number, was paginated independently, for its contents please turn to page 2 of this number.

Abrams, P., 17,80
Anastomosis workshop in Saudi Arabia, 49
Anderson, J. B., 26
Aneurysm of the interatrial septum and mitral valve prolapse, 40
Aziz, T. Z., 11
Barwell, N. J., 51
Bent Penis, management of, 79
Beynon, J., 26,78
Black, Michael, 38
Blundell, E., 77
Bone Marrow transplantation, 62
Book Reviews
  General Pathology, Walters and Isreal, 50
  Pocket Consultant Endocrinology, Hartog, 50
Bolton, R., 40
Bradfield, J. W. B., 78
Chadwick, D. J., 79
Channer, J. L., 78
Channer, K. S., 40
Charlton, Clive, 25
Children of the future age, 60
Clarke, Charles, 5
Clarke, T. J., 77
Computed Tomography of the internal auditory canals, 13
Computerised surgical audit, 52
Computers in surgery, 53
Crohn's Disease—a misnomer, 9
Crosfil, Martin, 34,51
Customer satisfaction in the surgical ward, 52
D. N. A. probes in lymphoreticular disease, 78
Dental postgraduate courses, trials and tribulations, 71
Desai, K. M., 79
Diagnostic imaging, expanding horizons, 61
Domestic Medicines, 34
Drowning, aspects of, 51
Eating tomorrow, 67
Epstein, M. A., 63
Feneley, R. C. L., 17
Ferro, M. A., 8
Fine needle aspiration biopsy in thyroid cancer, 26
Fine needle aspiration cytology of breast masses, 25
Gallstones, 25
Gartell, Paul, 25
Gaunt, P. N., 78
Gillatt, D. A., 52, 80
Gingell, J. C., 52, 79
Glover, S. C., 44
Goddard, P., 73
Goepel, J., 78
Grattan, C. E. H., 44
Harmer, Michael, 9
Harvey Cushing and Posterior Pituitary secretion, 11
Hasan, Syed S., 51
Heart and lung transplantation, 65
Heaton, K. W., 67
Hinchcliffe, A., 73
Hobbs, J. R., 62
Intestinal Fistula, 72
Irving, Miles, 72
James, I., 77
Jeans, W. D., 13
Kelly, Ciarnon, 38
Kennedy, C. T. C., 44
Kirollos, M. M., 79
Lateral thyroid ligament, 26
Lawrence, R. J., 53
Leaper, D. J., 49
Leow, C. K., 26
Levers, B. G. H., 71
Liver palpatation at laparotomy, 73
Lloyd, J. K., 60
Looking back to 1962, 51
Lucarotti, M. E., 17
Lumb, G. N., 79
Lymphoma and the kidney, 78
MacIver, A. G., 78
Madison, P. J., 77
Maitland, N., 78
Malpani, K. R., 73
Management of Diabetic Gangrene, 25
Mann, R. J., 44
Matthews, R. W., 71
Molecular Medicine, 64
Mortensen, N. J. McC., 26,78
Medical Education and Health Care, 5
Medulloblastoma with divergent glial differentiation, 77
Meningococcal meningitis, outbreak of, in the Royal Navy, 78
Morgan, Giles, 52
Nicoll, J. A. R., 77
O'Boyle, P. J., 79
Obituary, Dr Dennis Wright, 50
Perry, C. Bruce, 58
Primary care in the future, 59
Peyronies's disease, 79
Preventive Medicine in General Practice, 68
Prostatic cancer, new approaches to management of, 52
Prostatic disease, serum prostate specific antigen measurement in, 80
Rectal Carcinoma, in vivo staging with ulstasound, 26
Rectal tumour invasion, staging of, 78
Rees, J. Russell, 40
Rickett, John, 52
Rigby, H., 78
Rooney, N., 78
Russell, J., 78
Scully, C., 71
Shaw, J. F. L., 52
Shepherd, J. P., 71
Simcock, A. D., 51
Skerrett, F. D., 38
Smith, J. G., 78
Smith, M. A., 77
Smith, P. J. B., 80
Snow, M., 13
Southwood, W. F. W., 25
Stott, N. C. H., 59
Stower, M. J., 79,80
South West Urological Club, Meeting at Torbay, 1987, 79
Surgical Club of S. W. England, Meeting in Bath, 1986, 25
Surgical Club of S. W. England, Meeting in Truro and Penzance, 1987, 51
Surgical Safari, 48
Sweet's Syndrome and erythema nodosum, 44
T-Cell lymphocytosis and neutropenia, 77
Talc Pleurodesis in management of malignant pleural effusion, 51
Tawn, D. J., 13
Thomson, M. H., 26
Thomson, S., 73
Training in G. P. Surgeries, 38
Transplantation, retrieval of organs for, 52
Two hundred and fifty years of patient care at the B.R.I., 58
Turnbull, A. R., 25
Ultrasound scanning in urology outpatients, 79
Urethral Strictures, iatrogenic, 51
Urinary fibrinolysis and stone formation, 25
Urine Flow Clinic, A., 17
Urology diagnostic index, 80
Urostomy, practical problems with, 79
Urothelium, response to catheterisation, 77
Vesey, S. G., 79
Virus induced cancers, vaccine control of, 63
Vowles, Keith, 49
Wallwork, J., 65
Weatherall, Sir D. J., 64
Webb, A. J., 26
Wells, P. N. T., 61
Wessex Branch of the Association of Clinical Pathologists, Meeting in Bristol, 1987, 79
Wilde, P., 40
Williams, Jonathan, 68
Wooding, Regan, 38

